# Comment on Kopańska et al. Disorders of the Cholinergic System in COVID-19 Era—A Review of the Latest Research. *Int. J. Mol. Sci.* 2022, *23*, 672

**DOI:** 10.3390/ijms23052818

**Published:** 2022-03-04

**Authors:** Concetta Cafiero, Alessandra Micera, Agnese Re, Beniamino Schiavone, Giulio Benincasa, Raffaele Palmirotta

**Affiliations:** 1 Anatomic Pathology Unit, Fabrizio Spaziani Hospital, 03100 Frosinon, Italy; concettacafiero@gmail.com; 2 Research Laboratories in Ophthalmology, IRCCS—Fondazione Bietti, 00184 Rome, Italy; 3 Department of Chemistry and Clinical Biochemistry, Catholic University of Sacred Heart, Fondazione Policlinico Universitario Agostino Gemelli IRCCS, 00168 Rome, Italy; agnese.re@unicatt.it; 4 General Management Unit, Pineta Grande Hospital, 81030 Castel Volturno, Italy; beniamino.schiavone@pinetagrande.it; 5 Department of Clinical Pathology and Molecular Biology, Pineta Grande Hospital, 81030 Castel Volturno, Italy; giulio.benincasa@pinetagrande.it; 6 Interdisciplinary Department of Medicine, School of Medicine, University of Bari ‘Aldo Moro’, 70124 Bari, Italy

We read the recent review article by Marta Kopańska et al. titled “the Disorders of the Cholinergic System in COVID-19 Era—A Review of the Latest Research” [1]. First of all, we would like to congratulate the authors for the accurate revision of literature about COVID-19 and the cholinergic system [1]. The authors focused on the impairments of the cholinergic system, reporting evidence of dysregulation in acetylcholine (ACh)/acetylcholinesterase (AChE) activities and alteration in ACE2 circulating levels, as observed in myasthenia gravis, ocular myasthenia gravis, noxious skeletal muscles and multisystem inflammatory syndrome [1]. In this context, we would point out a few recent publications that might provide additional value to this interesting revision of the literature.

First, the author stated that “The SARS-CoV-2 spike protein has a sequence like neurotoxins, capable of binding nicotinic acetylcholine receptors (nAChR). This may be proof that SARS-CoV-2 can bind nAChR” [1]. The authors referred to the studies from Farsalinos and coworkers on the presence of a region of homology between neurotoxin and SARS-CoV-2 sequences [2]. We recently explored this aspect by confirming this homology and showing additional regions of homology between neurotoxin and SARS-CoV-2 sequences, which are adjacent to those previously reported [3]. Subsequently, we extended the analysis to the presence of different affinity regions and hypothesized that the RNA polymerase might be directly involved in this production, as we mentioned a “jumping mechanism” inside the last ORF of viral genome for spike glycoprotein, an aspect common to other viruses [3,4,5]. Due to this 50–60% homology with conotoxins, these small peptides might be responsible for the clinical manifestations (neurological, hemorrhagic and thrombotic) observed in COVID-19 patients [3,6,7,8,9,10]. This point has been recently sustained by the presence of circulating and local toxic products [3,9,10].

Second, the authors stated “there are many promising therapies that will prevent the SARS-CoV-2 virus from binding to the nicotinic receptor,” implying the possibility of new and attractive therapeutic and perhaps preventive implications [1,9,10]. To those therapies targeting the cholinergic system, we would point out the observations that elderly subjects develop more severe forms of disease or the worsening of disease in some youngsters [2,3]. Changes in microbiota upon aging or the presence of dysbiosis and/or certain tissue restricted bacterial profiles might account at least in part for these observations [11,12,13,14,15,16,17]. A correlation between specific nasopharyngeal/gut microbiota and disease severity has been previously reported, implying a probable participation of mucosal microbiota (nose, gut, conjunctiva) in some COVID-19 manifestations [11,12,13]. These observations encourage the latest hypothesis on the “bacteriophage-like” behavior of SARS-CoV-2 [14,18,19].

Overall, it is noteworthy to mention that multiple cholinergic receptors (cholinergic innervation) are expressed by ocular tissues [6]. As we cannot exclude that SARS-CoV-2 virus can also take advantage of the eye to enter the body, as ACE-2 receptors are expressed by mucosal conjunctiva as well as by some retinal subpopulations, and due to a peculiar microbiota populating the ocular surface, it is of great interest to highlight another article reporting that the omicron variant can bind ACE-2 receptors better than the Beta and Delta variants. This aspect would support higher diffusion and, in particular, more incidents of conjunctivitis, which be of some interest in any attempt to control the spread of the disease [7,8].

## Data Availability

Not applicable.

## References

[1] Kopańska M., Batoryna M., Bartman P., Szczygielski J., Banaś-Ząbczyk A. (2022). Disorders of the Cholinergic System in COVID-19 Era—A Review of the Latest Research. Int. J. Mol. Sci..

[2] Farsalinos K., Eliopoulos E., Leonidas D.D., Papadopoulos G.E., Tzartos S., Poulas K. (2020). Nicotinic cholinergic system and COVID-19: In silico identification of an interaction between SARS-CoV-2 and nicotinic receptors with potential therapeutic targeting implications. Int. J. Mol. Sci..

[3] Cafiero C., Micera M., Re A., Postiglione L., Cacciamani A., Schiavone B., Benincasa G., Palmirotta R. (2021). Could Small Neurotoxins-Peptides be Expressed during SARS-CoV-2 Infection?. Curr. Genom..

[4] Sola I., Almazán F., Zúñiga S., Enjuanes L. (2015). Continuous and discontinuous RNA synthesis in coronaviruses. Annu. Rev. Virol..

[5] Romano M., Ruggiero A., Squeglia F., Maga G., Berisio R. (2020). A structural view of SARS-CoV-2 RNA replication machinery: RNA synthesis, proofreading and final capping. Cells.

[6] Sastry B.V. (1985). Cholinergic systems and multiple cholinergic receptors in ocular tissues. J. Ocul. Pharmacol..

[7] Leonardi A., Rosani U., Brun P. (2020). Ocular Surface Expression of SARS-CoV-2 Receptors. Ocul. Immunol. Inflamm..

[8] Kumar S., Thambiraja T.S., Karuppanan K., Subramaniam G. (2022). Omicron and Delta variant of SARS-CoV-2: A comparative computational study of spike protein. J. Med. Virol..

[9] Li C.X., Chen J., Lv S.K., Li J.H., Li L.L., Hu X. (2021). Whole-transcriptome RNA sequencing reveals significant differentially expressed mRNAs, miRNAs, and lncRNAs and related regulating biological pathways in the peripheral blood of COVID-19 patients. Mediat. Inflamm..

[10] Petrillo M., Brogna C., Cristoni S., Querci M., Piazza O., Van den Eede G. (2021). Increase of SARS-CoV-2 RNA load in faecal samples prompts for rethinking of SARS-CoV-2 biology and COVID-19 epidemiology. F1000 Research.

[11] Bischoff S.C. (2016). Microbiota and aging. Curr. Opin. Clin. Nutr. Metab. Care.

[12] Li J.J., Yi S., Wei L. (2020). Ocular microbiota and intraocular inflammation. Front. Immunol..

[13] Zhang D., Li S., Wang N., Tan H.Y., Zhang Z., Feng Y. (2020). The cross-talk between gut microbiota and lungs in common lung diseases. Front. Microbiol..

[14] Ventero M.P., Cuadrat R.R.C., Vidal I., Andrade B.G.N., Molina-Pardines C., Haro-Moreno J.M., Coutinho F.H., Merino E., Regitano L.C.A., Silveira C.B. (2021). Nasopharyngeal microbial communities of patients infected with SARS-CoV-2 that developed COVID-19. Front. Microbiol..

[15] Ferreira C., Viana S.D., Reis F. (2020). Gut Microbiota dysbiosis-immune hyperresponse-inflammation triad in coronavirus disease 2019 (COVID-19): Impact of pharmacological and nutraceutical approaches. Microorganisms.

[16] De Oliveira G.L.V., Oliveira C.N.S., Pinzan C.F., De Salis L.V.V., Cardoso C.R.B. (2021). Microbiota modulation of the gut-lung axis in COVID-19. Front. Immunol..

[17] Di Stadio A., Costantini C., Renga G., Pariano M., Ricci G., Romani L. (2020). The microbiota/host immune system interaction in the nose to protect from COVID-19. Life.

[18] Sundararaman A., Ray M., Ravindra P.V., Halami P.M. (2020). Role of probiotics to combat viral infections with emphasis on COVID-19. Appl. Microbiol. Biotechnol..

[19] Kim D., Lee J.Y., Yang J.S., Kim J.W., Kim V.N., Chang H. (2020). The architecture of SARS-CoV-2 transcriptome. Cell.

